# On the Fly: Recent Progress on Autophagy and Aging in Drosophila

**DOI:** 10.3389/fcell.2019.00140

**Published:** 2019-07-24

**Authors:** Tamás Maruzs, Zsófia Simon-Vecsei, Viktória Kiss, Tamás Csizmadia, Gábor Juhász

**Affiliations:** ^1^Institute of Genetics, Biological Research Centre of the Hungarian Academy of Sciences, Szeged, Hungary; ^2^Department of Anatomy, Cell and Developmental Biology, Eötvös Loránd University, Budapest, Hungary

**Keywords:** aging, autophagy, Drosophila, dietary restriction (DR), spermidine

## Abstract

Autophagy ensures the lysosome-mediated breakdown and recycling of self-material, as it not only degrades obsolete or damaged intracellular constituents but also provides building blocks for biosynthetic and energy producing reactions. Studies in animal models including Drosophila revealed that autophagy defects lead to the rapid decline of neuromuscular function, neurodegeneration, sensitivity to stress (such as starvation or oxidative damage), and stem cell loss. Of note, recently identified human Atg gene mutations cause similar symptoms including ataxia and mental retardation. Physiologically, autophagic degradation (flux) is known to decrease during aging, and this defect likely contributes to the development of such age-associated diseases. Many manipulations that extend lifespan (including dietary restriction, reduced TOR kinase signaling, exercise or treatment with various anti-aging substances) require autophagy for their beneficial effect on longevity, pointing to the key role of this housekeeping process. Importantly, genetic (e.g., *Atg8a* overexpression in either neurons or muscle) or pharmacological (e.g., feeding rapamycin or spermidine to animals) promotion of autophagy has been successfully used to extend lifespan in Drosophila, suggesting that this intracellular degradation pathway can rejuvenate cells and organisms. In this review, we highlight key discoveries and recent progress in understanding the relationship of autophagy and aging in Drosophila.

## Introduction

The search for eternal youth has always been a central idea in the history of mankind, as aging and ultimately death represents a startling and inevitable fate for all living organisms. Although the philosophers’ stone has not been found yet, it can be considered one of the major achievements of our civilization that human life expectancy has been rising by 2.5 years/decade in a linear manner from the middle of the 19th century (Oeppen and Vaupel, 2002). However, the increasing proportion of older individuals in our society raises problems that come from the nature of aging, i.e., the functional decline in the health of the elderly. Strikingly, aging is the main risk factor for the major chronic and killer diseases such as dementia, cardiovascular disease, and cancer (Niccoli and Partridge, 2012), and the prevalence of these diseases increases nearly exponentially with age. As taking care of diseased elderly represents a significant social and economic burden on society, there is increasing pressure on biomedical scientists to come up with ways of extending healthspan along with increased lifespan (Goldman et al., 2013).

To intervene in the process of aging, the first step would be to elucidate the mechanisms underlying aging, and then to find ways of maintaining health through the manipulation of these mechanisms (Partridge, 2014). We still do not know how cellular function is exactly disrupted during aging, but we can obviously see the complexity of the process as it tends to affect practically all organs and tissues. By looking at it from a bird’s eye view, it seems to be an almost impossible quest to pinpoint one single cause underlying the aging process or to ameliorate all aspects of aging simultaneously in various tissues. However, the correlation of age with the increased prevalence of diseases with seemingly different pathological features may suggest the existence of common background mechanisms. Importantly, several features of aging on the level of individual organs and tissues can be deduced from compromised cell functions. As an attempt to give a conceptual framework for the field of aging research, these characteristics have recently been summarized as the nine hallmarks of aging (López-Otín et al., 2013). Acknowledging that these hallmarks are interconnected, the authors grouped them into three hierarchical categories: (1) primary hallmarks (2), antagonistic hallmarks and (3) integrative hallmarks. As the first category, primary hallmarks of aging have unequivocally negative effects on normal cellular function and are considered to be the main causes of cellular damage. These five hallmarks are genomic instability, telomere erosion, epigenetic alterations, mitochondrial dysfunction, and loss of proteostasis. Damage accumulated through these processes triggers compensatory responses representing the second category, the two antagonistic hallmarks of aging: altered nutrient sensing and cellular senescence. These mechanisms initially mitigate the damage, but upon reaching a chronic level they subvert their purpose and become deleterious themselves. For example, cellular senescence at low level can protect the organism from cancer (by blocking the proliferation of damaged and potentially oncogenic cells), but may promote aging after reaching a higher intensity. Ultimately, damage progressively accumulated by the primary and antagonistic hallmarks will overload the compensatory capacity of cellular homeostasis and manifest in the third category, the two integrative hallmarks of aging. These are stem cell exhaustion and altered intercellular communication (most prominently a chronic, systemic inflammation) which are eventually responsible for the functional decline during aging.

Besides the description of the characteristics of the aging process, the most significant finding of aging research is that aging now is considered to be a malleable process (Gems and Partridge, 2013). The lifespan of organisms can be extended by both environmental and genetic traits and more importantly, their healthspan can simultaneously be improved during aging, which shows that it is possible to reach the ultimate goal of aging research. Recently, a growing body of evidence shows that alterations of autophagy, the main self-degradative process of eukaryotic cells, likely plays a central role in the aging process. Observations in various organisms indicate that aging and autophagy have a bidirectional connection with each other (Rubinsztein et al., 2011; Hansen et al., 2018). On one hand, autophagic degradation shows an age-dependent decline and impairment of autophagy contributes to the development of age-associated diseases (Lipinski et al., 2010; Carnio et al., 2014; Chang et al., 2017). On the other hand, lifespan-extending interventions largely depend on the autophagy machinery for their beneficial effects on longevity (Tóth et al., 2008; Alvers et al., 2009; Eisenberg et al., 2009). Strikingly, multiple longevity pathways seem to converge on autophagy, and genetic or environmental factors that affect lifespan through these pathways at least partly exert their effects via the modulation of autophagy. These observations point to the key role of autophagy in aging, and suggest that increased autophagy may compensate for at least some of the cellular hallmarks of aging. Thus, the housekeeping functions of autophagy can counteract the accumulation of cellular damage, which is considered to be a primary mechanism driving aging.

The fact that the aging process and its hallmarks are similar in all organisms points to its strong evolutionary conservation, and offers a unique way to understand the aging process by observations made in various model organisms (Gems and Partridge, 2013). Amongst experimental animals, research in the fruit fly *Drosophila melanogaster* contributed greatly to our understanding of the underlying mechanisms of both aging and autophagy (Mulakkal et al., 2014; Hansen et al., 2018). In this review, we give a brief historical outline of the milestones reached in these increasingly interweaving research fields and highlight key discoveries made in Drosophila. We also give an overview of how aging and age-related diseases manifest in fly tissues and how these phenomena connect to autophagy. We summarize the cellular signaling networks that regulate aging and autophagy and the established ways of modulating these processes, including pharmacological interventions that may counteract aging at least partly via the modulation of autophagy.

## Drosophila as a Model to Study Autophagy and Aging

### Historical Overview of Aging Research and the Main Mechanisms Involved in the Regulation of Lifespan

Fasting has been practiced for millennia mainly due to religious reasons, and the first scientific report showing that reduced food intake can prolong lifespan was published in 1935 (McCay et al., 1989). McCay et al. (1989) showed that dietary restriction (DR) extends the lifespan of laboratory rats in the first scientifically accurate observation of the effect of an environmental factor on longevity, which represents the beginning of aging research. Although the effect of DR on lifespan was successfully reproduced in various model and non-model organisms, it took another 48 years to find the first evidence that genetic factors are also involved in the regulation of lifespan. The first single-gene mutation leading to extended lifespan was the *daf-2* mutant strain of *C. elegans* (Klass, 1983), and this discovery represents the first mechanistic connection between nutrient sensing and longevity: the protein encoded by *daf-2* turned out to be the single insulin/insulin-like growth factor receptor of the worm (Kenyon et al., 1993; Kimura et al., 1997). The central and evolutionarily conserved role of this signaling pathway in longevity was firmly established in the following years, as further members of the pathway were also identified through the isolation of long-lived mutants of *C. elegans* and other laboratory organisms as well. In Drosophila, the first such long-lived mutants were a heteroallelic combination of hypomorphic insulin receptor gene alleles, and null mutants of the *chico* gene encoding the single Drosophila insulin receptor substrate protein (Clancy et al., 2001; Tatar et al., 2001). Importantly, mice mutant for members of this pathway were also found to be long-lived (Holzenberger et al., 2003; Selman et al., 2008), and natural genetic variants of the components of the pathway turned out to be associated with longevity in humans (Pawlikowska et al., 2009).

Strikingly, these studies also showed that both DR- and mutation-induced lifespan extensions are accompanied by a broad spectrum of health improvements involving tissues that seemingly do not have any connection with each other. This shows the malleability of aging and presumes the existence of mechanisms that mediate these beneficial effects. Decreased activity of TOR kinase is likely crucial for mediating such effects, because mutations in the components of this pathway increase lifespan in various organisms including humans (Jia et al., 2004; Kapahi et al., 2004; Kaeberlein et al., 2005; Passtoors et al., 2013). The TOR complex integrates cellular nutritional and other signals, and acts as the central positive regulator of growth and inhibitor of autophagy. Under nutrient-scarce conditions, reduced TOR activity leads to downregulation of cap-dependent protein synthesis and unleashes autophagy, thus liberating building blocks and energy from autophagic cargo. Strikingly, multiple drugs with lifespan-extending effects modulate the TOR pathway and require autophagy for their beneficial effects. These observations point to a central role of the insulin/TOR signaling network and autophagy in promoting longevity.

### Historical Overview of Autophagy Research and the Process of Autophagy

The history of autophagy research can be divided into two major chronological periods (Mulakkal et al., 2014). Studies of the early era that were primarily based on ultrastructural analyses mainly provided descriptive data. This approach was somewhat superseded by the tools of the genomic era allowing mechanistic analysis and the use of specific marker proteins. The first properly interpreted electron microscopic images of mitochondria-containing lysosomes were taken in the proximal tubule cells of rat kidney by Novikoff (1959), and during the next decades, ultrastructural analyses in various tissues revealed the morphological stages of the autophagic process. The first observations that pointed to a connection between the insulin pathway and autophagy showed that the process can be induced by glucagon and inhibited by insulin (Deter et al., 1967; Pfeifer, 1977). Drosophila was an important experimental organism even in the early era, and the first report of autophagic structures in larval fat body cells dates back to 1963 (Von Gaudecker, 1963). During the 1970s and 1980s, ultrastructural analyses showed that fat body cells accumulate large amounts of stored nutrients that can be mobilized through developmentally programmed autophagy controlled by the molting hormone ecdysone (Butterworth et al., 1988). This intensive catabolic process ultimately leads to the decomposition of this tissue, thus providing building blocks and energy for the metamorphosis of the fly larvae. The first genes involved in the molecular mechanism of autophagy were identified in yeast at the beginning of the 1990s (Tsukada and Ohsumi, 1993). This milestone revolutionized autophagy research and gave new momentum to the field. The fundamental importance of autophagy and the discovery of Atg (autophagy-related) genes were acknowledged with a Nobel-prize awarded to Yoshinori Ohsumi in 2016. Notably, Drosophila was the first animal in which an Atg gene was characterized. It was shown that the product of *draut1/Atg3* is essential for autophagy and proper development (Juhász et al., 2003). Since then, a still expanding genetic toolkit and sophisticated experimental approaches have been developed to study autophagy in Drosophila (Mauvezin et al., 2014; Nagy et al., 2015; Lőrincz et al., 2017; Takáts et al., 2019). Interestingly, both starvation-induced and ecdysone-mediated developmental autophagy turned out to be initiated by the downregulation of the insulin signaling pathway (Rusten et al., 2004; Scott et al., 2004). Importantly, an early study showed that loss of Atg7 decreases lifespan in Drosophila (Juhász et al., 2007a).

These results suggested a connection between insulin signaling, autophagy and longevity and in the last decade, several key discoveries of this field were achieved by using the Drosophila model. Systemic effects of tissue-specific modulation of autophagy on organismal aging were first observed in the fruit fly: neuronal overexpression of *Atg8a* or *Atg1* were shown to be sufficient to extend lifespan (Simonsen et al., 2008; Ulgherait et al., 2014), and the overexpression of *Atg1* also improved intestinal barrier function. The loss of proteostasis during muscle aging was linked to decreased autophagy and organismal aging (Demontis and Perrimon, 2010), and the requirement for autophagy was shown in a conserved longevity paradigm involving mTOR inhibition in the fly model (Bjedov et al., 2010). Importantly, a direct connection between Parkin, a key regulator of mitophagy and longevity was established by the discovery that *parkin* overexpression is sufficient to extend Drosophila lifespan (Rana et al., 2013).

Three main mechanisms of autophagy have been described through which cytoplasmic components can reach lysosomes. During chaperone-mediated autophagy, proteins with an exposed KFERQ-like motif are recognized by the Hsc70 chaperone and then targeted to the transmembrane LAMP2a protein located in the lysosomal membrane (Cuervo and Wong, 2014). With the possible contribution of other chaperones, proteins are translocated into the lysosomal lumen through the channel formed by a LAMP2a oligomer. The second type of autophagy that is also linked directly to the lysosomal membrane is microautophagy, during which cytoplasmic components are directly taken up by lysosomes. This way, portions of the cytoplasm and even whole organelles are surrounded by invaginations of the lysosomal membrane, which will eventually pinch off into the lumen of the lysosome (Li et al., 2012; Tekirdag and Cuervo, 2018). Bulk and selective types are known in the case of both micro- and macroautophagy. Macroautophagy is the best known autophagy pathway and it significantly differs from chaperone-mediated and microautophagy in terms of its mechanisms (Feng et al., 2014). Macroautophagy requires the formation of a new organelle: the autophagosome, in which cytoplasmic components are sequestered prior to their degradation by the lysosome. As the first step of this process, a membrane cistern called the phagophore (or isolation membrane) forms in the cytoplasm. The growing phagophore surrounds a portion of the cytosol, and the sealing of its edges leads to the formation of the double-membrane autophagosome. This process depends on the evolutionarily conserved set of Atg genes (Table 1). The outer membrane of this transient vesicular organelle then fuses with lysosomes (or first with late endosomes) to give rise to autolysosomes (or amphisomes), in which lysosomal degradation of the inner autophagosomal membrane as well as the cargo takes place. While macroautophagy has originally been considered to be a bulk, non-selective process, several subtypes of selective autophagy have been described by now that transport particular structures for lysosomal degradation including pexo-, mito-, aggre-, reticulo-, lipo-, and ribophagy (Rogov et al., 2014). Amongst the three types, macroautophagy (hereafter referred to as autophagy) has the largest capacity and is considered to be the main clean-up process of eukaryotic cells. Importantly, a basal level of autophagy proved to be essential in maintaining normal cell function through the removal of damaged and obsolete cellular components (Klionsky and Codogno, 2013). In addition, autophagy is known to be induced by starvation or other stresses that cells encounter, which often signals through the downregulation of TOR kinase.

**Table 1:** Drosophila autophagy-related genes and their functional analyses.

Atg proteins in *Drosophila*	Function	Loss of function phenotype	Gain of function phenotype	References
Atg1	Serine/threonine kinase	Short lived, oxidative stress response defects, reduced dendritic growth, autophagy defect	Extended lifespan, improved intestinal barrier, reduced cell size, induced autophagy, induction of apoptosis, and organismal death upon strong overexpression	Scott et al., 2004, 2007; Wu et al., 2009; Pircs et al., 2012; Chang et al., 2013; Nagy et al., 2014b; Ulgherait et al., 2014; Pérez et al., 2015; Clark et al., 2018
Atg2	Lipid transporter for autophagosome biogenesis	Short lived, decreased cell death, reduced dendritic growth, autophagy defect	–	Scott et al., 2004; Pircs et al., 2012; Chang et al., 2013; Nagy et al., 2014a; Xu et al., 2015; Clark et al., 2018; Valverde et al., 2019
Atg3 (aka. Aut1)	E2-like enzyme, Atg8 ligase activity	Short lived, autophagy defect	–	Juhász et al., 2003; Scott et al., 2007; Chang et al., 2013; Pérez et al., 2015; Varga et al., 2016
Atg4a	Cysteine-type endopeptidase	Accumulation of p62 positive aggregates, autophagy defect	–	Pircs et al., 2012; Pérez et al., 2015
Atg4b	Cysteine-type endopeptidase	–	–	–
Atg5	Atg8 ligase activity	Memory defect, autophagy defect, ataxia, reduced dendritic growth, impaired immune function	–	Scott et al., 2004; Ren et al., 2009; Chang et al., 2013; Pérez et al., 2015; Kim et al., 2016; Clark et al., 2018; Bhukel et al., 2019
Atg6	Autophagosome assembly, late endosome-lysosome maturation	Autophagy defect, melanotic mass formation	–	Chang et al., 2013; Shravage et al., 2013; Lõrincz et al., 2014; Pérez et al., 2015
Atg7	E1-type ligase, Atg8 and Atg12 activator enzyme	Short lived, memory defect, impaired immune function, autophagy defect	Extended lifespan	Scott et al., 2004; Juhász et al., 2007a; Ren et al., 2009; Chen et al., 2012; Erdi et al., 2012; Pircs et al., 2012; Chang et al., 2013; Nagy et al., 2014a; Pérez et al., 2015; Varga et al., 2016
Atg8a	Ubiquitin like protein, autophagosome assembly	Reduced dendritic growth, autophagy defect	Extended lifespan	Scott et al., 2007; Simonsen et al., 2008; Pircs et al., 2012; Chang et al., 2013; Nagy et al., 2014a; Ulgherait et al., 2014; Pérez et al., 2015; Clark et al., 2018
Atg8b	Ubiquitin like protein	–	–	Simonsen et al., 2008; Pérez et al., 2015
Atg9	Organization of PAS, response to oxidative stress, JNK activation	Thicker and shorter midgut, memory defect, autophagy defect, short lived	Induced JNK activation	Pircs et al., 2012; Tang et al., 2013; Nagy et al., 2014a; Pérez et al., 2015; Wen et al., 2017; Bhukel et al., 2019
Atg10	E2-like enzyme, Atg12 transferase activity	–	–	–
Atg12	Ubiquitin like protein, Atg8 ligase activity	Reduced immune function, autophagy defect	–	Scott et al., 2004; Ren et al., 2009; Chang et al., 2013; Pérez et al., 2015
Atg13	Atg1 regulator activity	Short lived, autophagy defect	–	Chang and Neufeld, 2009; Pircs et al., 2012; Chang et al., 2013; Nagy et al., 2014b; Katheder et al., 2017; Melani et al., 2017
Atg14	Autophagosome assembly	Autophagy defect, decreased stem cell number in the gut	–	Pircs et al., 2012; Lõrincz et al., 2014; Nagy et al., 2016; Katheder et al., 2017
Atg16	Atg8 ligase activity	Defective immune responses, inflammation, thicker and shorter midgut, short lived, autophagy defect	–	Pircs et al., 2012; Chang et al., 2013; Varga et al., 2016; Nagy et al., 2017
Atg17 (aka. FIP200)	Atg1 regulator activity	Short lived, autophagy defect	Enhanced autophagy	Kim et al., 2013; Nagy et al., 2014b

## Characteristics of Aging Drosophila Tissues and Their Contribution to Organismal Aging

Aging manifests in several ways on the cellular and organismal level and age-associated phenotypes are seen in various tissues, which ultimately lead to the development of diseases linked to aging. There is a growing body of evidence (see below) supporting correlations between the aging rate of a given tissue and that of the whole organism. However, we still do not know whether it is the aging of a single, critical tissue (e.g., the brain or the gut) that determines aging rate on the organismal level or if it arises from the combination of defects affecting multiple tissues. In this chapter we highlight some tissues that may have such rate-limiting properties during aging.

### Fat Tissue

As the equivalent of mammalian hepatocytes and white adipocytes, the main function of the Drosophila fat tissue is the uptake, metabolism and storage of nutrients that enter the hemolymph after initial processing in the gut. During the development of the almost continuously feeding fly larva, fat body cells undergo an enormous, more than 200-fold increase in size (accompanied by a 256–512 fold polyploidization) thanks to intensive nutrient storage in the cytoplasm. Importantly, this anabolic process can be transiently reversed by starvation, during which the induction of autophagy liberates nutrients from fat cells to the hemolymph to ensure the survival of the diploid larval tissues (Mulakkal et al., 2014). These tissues will give rise to the organs of adult flies in parallel with the breakdown of polyploid larval organs during metamorphosis. The disintegration of these polyploid tissues (including the fat body, gut and salivary glands) is initiated by a peak of the molting hormone ecdysone, and the process is executed by a massive, irreversible autophagy induction in fat and gut cells. The fat tissue initially decomposes to individual cells, half of which die during metamorphosis and their liberated components are used as building blocks and to provide energy for the development of adult tissues. Notably, as dying fat cells are full of autophagic structures, this process was often referred to as programmed cell death type II or autophagic cell death (Butterworth and Forrest, 1984). Interestingly, the other half of the larval fat cells survive to the first days of adulthood and die through caspase-dependent cell death (Aguila et al., 2007). This self-sacrifice nicely demonstrates the central role of fat cell autophagy in the nutrient handling and allocation during the postembryonic development of Drosophila.

Importantly, a picture is emerging about a more complex role of fat tissue beyond providing nutrients for other tissues through self-cannibalism. Besides being an important storage organelle, the fat tissue also acts as a main sensor of nutrients and a central regulator of systemic insulin signaling and longevity (Tatar et al., 2014). It has been shown previously that inhibition of amino acid transport specifically in the fat tissue leads to reduced organismal growth (Colombani et al., 2003), and the molecular components and main mechanisms of this effect were identified in the past decade. The mediators of this regulatory network are humoral factors: the Drosophila insulin-like peptides (DILPs). These proteins are secreted by fat cells and 14 neurosecretory insulin-producing cells (IPCs) located in the larval and adult brain. Based on sequence similarity, seven Drosophila *dilp* loci have been identified (Brogiolo et al., 2001; Cao and Brown, 2001). Expression of *dilp1, dilp2, dilp3*, and *dilp5* is high in IPCs whereas *dilp6* is highly expressed in the fat tissue. During starvation, reduced TOR signaling leads to FOXO activation and in turn to the increased expression and secretion of DILP6 in fat cells. Circulating DILP6 then causes the retention of DILP2 and DILP5 proteins in IPCs (that may not be capable of nutrient sensing on their own), and reduced DILP secretion leads to organism-wide reduction of insulin-signaling and extended lifespan (Géminard et al., 2009; Bai et al., 2012). Thus, the reduced level of nutrients in the hemolymph induces autophagy in fat cells in a cell-autonomous manner, and fat cells downregulate systemic insulin signaling cell-non-autonomously that results in restricted growth and extended lifespan.

Recently, another non-cell-autonomous function of fat tissue was shown to have an effect on aging. Badinloo et al. (2018) showed that in the fat body cells of the adult flies, there is a strong correlation between the aging process and the activation of immune-related genes. The expression levels of the NF-κB like transcription factor *Relish* and its target genes encoding antimicrobial peptides (AMPs) such as *attacins (A, C, D), cecropinA1, defensin, diptericin, drosocin, drosomycin*, and *metchnikowin* are increased during the aging process in the adult fat body. The fat body specific overexpression of *Relish* led to increased levels of AMPs, which strongly correlated with shortened Drosophila adult lifespan. Furthermore, the reduced expression of *Relish* decreased the levels of AMPs and these genetically manipulated animals showed an extended lifespan. These findings show that the activation of immune related genes positively correlates with aging phenotypes in fat cells of adult flies, which is the hallmark of the age-associated immune response. The authors also showed that overexpression of individual AMPs such as *attacinA, cecropinA1, defensin* and *metchnikowin*, but not *drosocin* and *drosomycin*, may contribute to shorter lifespan, possibly via cytotoxicity and higher apoptotic levels. But how can these factors promote aging and apoptosis, and why is autophagy important in the prevention of this process? One could speculate that AMPs cause mitochondrial depolarization and lead to apoptosis in fat cells, contributing to the aging phenotype this way. Autophagy is important in the recognition, sequestration and elimination of damaged mitochondria (Palikaras et al., 2018; Rongvaux, 2018; Picca et al., 2019), and high levels of mitophagy may protect the adult fat cells from early aging and apoptosis.

Oenocytes also fulfill liver-like functions in Drosophila and show interesting biochemical and molecular changes during aging (Huang K. et al., 2019). In this article, the authors identified the main molecular alterations of aging oenocytes using cell type-specific ribosome profiling (RiboTag). They find that some of the age-regulated genes that are involved in oxidative phosphorylation, fatty acid metabolism, proteasome function and cytochrome P450 pathways are downregulated in aged oenocytes. Interestingly, the levels of most peroxisomal genes such as components of fatty acid β-oxidation and peroxisome biogenesis also decreased. In contrast, the immune-response pathways, Ras/MAPK signaling and DNA replication are upregulated in this condition. These findings suggest that certain molecular alterations greatly contribute to the aged cell phenotype.

### Intestine

The gastrointestinal tract is a very important and specialized microenvironment in the animal body. In the fly gut, nutrients from the digested food are absorbed via the intestinal epithelial cells (enterocytes), which utilize septate junctions to form an effective barrier against pathogens (Schulte et al., 2003; Apidianakis and Rahme, 2011; Lemaitre and Miguel-Aliaga, 2013). The intestine has diverse regions, which contain morphologically and histologically different epithelial cells in Drosophila. The main regions of the midgut (anterior, middle, and posterior) and further sub-regions can be distinguished from each other based on their different expression profiles (Apidianakis and Rahme, 2011; Miguel-Aliaga et al., 2018). The intestine also has other important functions such as metabolism and immune homeostasis (Jasper, 2015).

The turnover and quick regeneration of the intestinal epithelium is ensured by intestinal stem cells. Maintenance of epithelial integrity and barrier function in the gut via the activity of intestinal stem cells is absolutely critical for longevity. During aging, the barrier function of intestinal epithelia deteriorates, which is detrimental for Drosophila lifespan (Rera et al., 2012; Clark et al., 2015). The composition of the intestinal microbiota also dramatically changes during aging. Expansion of several bacteria such as Gamma-proteobacteria is associated with the breakdown of the epithelial barrier via the activation of an intestinal immune response (Clark et al., 2015).

One of the main mechanisms that maintains intestinal stem cell functions and homeostasis is autophagy. Autophagy dysfunction during aging can lead to increased DNA damage, cell cycle arrest and apoptosis (Nagy et al., 2018). After an initial hyperplasia phenotype (Tusco et al., 2017), the thinning of the intestinal wall is seen over time in most Atg gene mutants and RNAi animals. However, the midguts of *Atg9, Atg16*, and *Atg101* mutant flies are much thicker and shorter compared to controls, which is likely at least in part due to altered TOR kinase signaling and differentiation defects of intestinal stem cells, respectively (Nagy et al., 2017; Wen et al., 2017; Guo et al., 2019).

### Immune Responses

Although the number of infection-related deaths are decreasing among the elderly in well-developed Western societies, these still remain a major factor in determining longevity. An elegant study recently showed that stochastic variation in immune responses determines the survival of isogenic, co-housed Drosophila adults upon infection (Duneau et al., 2017). The central importance of stochastic variation in life or death decisions is rather unappreciated, but it is likely of general importance in aging-related mortality as well.

### Muscle

In Drosophila, the structure and function of skeletal muscle fibers is similar to that of mammals. During aging, the decay of muscle function presents as decreased muscle strength (muscle quality) and reduction in total muscle mass (muscle quantity) known as sarcopenia (Demontis et al., 2013). Unlike in mammals, muscle stem cells called satellite cells have not been identified in adult insects, so fly skeletal muscle cells are not replenished during aging and thus undergo a more dramatic age-related decline. This fundamental difference explains the more apparent morphological and functional alterations in aging Drosophila skeletal muscle compared with mammals, and it is supposedly related to the relatively short lifespan of flies (Demontis et al., 2013). During aging, muscle cells accumulate malfunctioning mitochondria and protein aggregates as a consequence of imbalanced proteostasis, which both contribute to muscle dysfunction. Importantly, autophagy selectively removes both protein aggregates and dysfunctional mitochondria, so its decline is likely a major reason for aging-related changes.

In line with this, loss of the transmembrane protein Atg9 or its partners Atg2 or Atg18 in heart and flight muscles caused shortened lifespan in Drosophila. The knockdown flies showed cardiac hypertrophy and structural abnormalities in the heart, resembling the age-dependent decline of cardiac functions (Xu et al., 2019). In these autophagy defective heart cells, abnormal elongated mitochondria were observed and the number of mitochondria captured in autophagosomes was reduced. Interestingly, the flight muscle cells contained fewer mitochondria that were often fragmented.

Aging of the skeletal muscle has a strong impact on nutrient sensing and global aging in part because myokines, muscle-derived cytokines and growth factors, modulate systemic physiology and aging, so proper muscle function is critical for longevity (Demontis et al., 2013). In Drosophila, the skeletal muscle transcription factor Mnt induces myoglianin (Myo) production, which is a transforming growth factor-β (TGF-β) ligand, and it decreases nucleolar size and ribosomal RNA level in the fat body. Overexpression of both Mnt and Myo increase longevity of flies (Demontis et al., 2014) Besides this, muscle produces ImpL2 as well (the ortholog of human insulin-like growth factor-binding protein 7), which elevates the number of lysosomes and may promote increased autophagic flux in skeletal muscle. ImpL2 inhibits overall insulin signaling, thus it can contribute to the lifespan extension of flies through the above mentioned mechanisms (Owusu-Ansah et al., 2013).

### Nervous System

Neurons are specialized cells for integrating, processing and transmitting information. Our brain consumes 20% of total blood glucose even though its weight is only about 2.5% of that of our body, demonstrating how high the neuronal metabolic activity is. Besides these, neurons have specialized structures for information delivery: axons, dendrites, and synapses. Quality control of mitochondria and proteins in the axon and synapse is crucial for maintaining the proper functioning of neurons, and since these cells are post-mitotic, it predisposes them for the accumulation of defective organelles and toxic proteins. That is why autophagy plays a critical role in normal neural functions, and its failure leads to the accumulation of obsolete cellular components, which may result in the development of neurodegenerative diseases. The first report of an autophagy gene null mutant in Drosophila showed that loss of Atg7 leads to the accumulation and aggregation of undegraded proteins in the cytoplasm, premature neuronal aging and apoptosis, ataxia and short lifespan (Juhász et al., 2007a). Several other components of the autophagic machinery including Atg17/FIP200 (Kim et al., 2013), Atg8a (Simonsen et al., 2008), Atg9 (Wen et al., 2017), and Atg16 (Varga et al., 2016) were also found to be essential for the maintenance of proper neural functions and normal lifespan. Importantly, the neuron-specific overexpression of *Atg8a* was found to induce longevity, presumably through enhanced autophagy (Simonsen et al., 2008). SNARE proteins promote vesicle fusions, and by now the SNAREs necessary for autophagosome clearance are also known: SNAP-29, Vamp7 and the autophagosomal Syntaxin 17 (Syx17). In *Syx17* mutant flies, autophagosomes accumulate in all cells including neurons (Takáts et al., 2013), which also leads to neuromuscular dysfunction as indicated by their poor performance in climbing tests, and to early death of the animals.

A typical symptom of aging is memory loss, and a recent study reported that autophagy affects memory function in flies (Bhukel et al., 2019). Impaired autophagy due to silencing of *Atg5* or *Atg9* in the learning center (mushroom body) of the Drosophila brain attenuates associative olfactory memory and decreases the level of neuropeptide Y, which normally acts non-cell-autonomously to promote memory function. Thus, an autophagy-regulated signaling pathway promotes memory formation in flies.

Both normal and pathological, early aging of the nervous system are characterized by impaired energy metabolism, increased oxidative stress and accumulation of protein aggregates. This latter phenomenon is often investigated in Drosophila, which is a popular model for neurodegeneration. Autophagy plays a pivotal role in maintaining the integrity of postmitotic neurons, since in the absence of cell division, the damaged organelles and misfolded proteins cannot be “diluted.” Perturbed autophagy interferes with intracellular communication in neurons, during which organelles and proteins should be transported to relatively high distances from the perikaryon. In particular cases including Alzheimer’s and Parkinson’s disease, accumulation of disease-specific proteins (see below) can perturb autophagy, which leads to the accumulation of autophagosomes, and contributes to neurodegeneration (Son et al., 2012).

Importantly, age-related dementias (diseases with deterioration of mental abilities during aging) strongly affect life quality. Seventy-five percent of dementias are caused by Alzheimer’s disease, and other common types include vascular dementia and dementia with Lewy bodies (Dening and Sandilyan, 2015). Lewy bodies - composed of α-synuclein – can also be found in Parkinson’s disease, which eventually causes dementia, too. Mutations in the huntingtin gene (*HTT*) are responsible for the onset of Huntington’s disease (HD), and 50% of people with advanced HD develop dementia as well. Mixed typed dementias occur when the patient suffers from multiple different dementias; the most frequent is when Alzheimer’s disease and vascular dementia occur at the same time (Dening and Sandilyan, 2015).

Based on the estimation by the Alzheimer’s Society, 44 million people may be suffering from this disease worldwide (5–7% of all people above 60 years), and this number will double in every 20 years (Dening and Sandilyan, 2015). Approximately one third of the people with dementia live in care homes, the remainders often need a primary caregiver (mainly a family member), which means extra expenses for the individuals and for the society as well. Thus, developing new therapies, which target the causes of the diseases or slow their progression, is a pressing need.

The most challenging part of neurodegenerative disease research is the identification of the triggering causes, because approximately 90% of the Parkinson’s (Tas et al., 2018) and Alzheimer’s disease (Gatz et al., 2006) cases are sporadic without a clear genetic link. Although we are still far from understanding the molecular mechanisms of disease onset and progression, boosting the autophagic turnover of proteins and organelles will likely be beneficial for the treatment of dementia (please see the recent reviews: Boland et al., 2018; Hansen et al., 2018).

## Signaling Pathways Involved in the Regulation of Autophagy and Aging

### TOR Ser/Thr Kinase

Target of rapamycin (TOR) is a member of the phosphatidylinositol kinase-related kinase (PIKK) family. Based on the analysis of the interaction proteome of Drosophila TOR, two main complexes were identified: TORC1 and TORC2. TORC1 contains the fly orthologs of the mammalian TORC1 complex: TOR, Raptor, Lobe, Lst8 (dGbL) (Glatter et al., 2011). TORC1 as a signaling hub has a central role in nutrient-sensing, and hence in the positive regulation of cell growth and anabolic metabolism in eukaryotes. TORC2 is composed of TOR, SIN1, Lst8 and Rictor; it regulates actin polymerization and plays a role in age-associated memory loss of flies (Johnson et al., 2015). Hereafter we will focus on those functions of TOR that are mediated by the TORC1 complex. TOR kinase activity inhibits autophagy via direct phosphorylation of Atg1, the kinase involved in the initiation of autophagosome formation. TOR deficiency more than doubles the lifespan of *C. elegans* (Vellai et al., 2003), and TOR kinase inhibitors such as rapamycin enhance autophagy and promote longevity in flies, too (Bjedov et al., 2010). Along these lines, overexpression of the TOR kinase inhibitor TSC1 and TSC2 (tuberous sclerosis complex 1 and 2) proteins were found to increase the lifespan of flies (Kapahi et al., 2004).

### FOXO (Forkhead Box O) Transcription Factor

FOXO is activated by decreased growth/class I PI3K or increased stress (JNK) signaling through inhibitory and stimulatory phosphorylation events, respectively (Sun et al., 2017). FOXO has been shown to be required for growth inhibition, increased stress resistance, and lifespan extension triggered by modulation of these signaling pathways, respectively (Jünger et al., 2003; Wang et al., 2005). The first report of FOXO regulating autophagy came from a Drosophila study, which showed diminished starvation response in *foxo* mutant larvae and that overexpression of activated FOXO is sufficient to induce autophagy in fat cells of feeding larvae (Juhász et al., 2007b). This regulation turned out to be conserved in mammals, too (Mammucari et al., 2007).

The importance of FOXO for longevity was shown in 1983, as its worm homolog *daf-16* was necessary for *daf-2* mutation-induced longevity (Klass, 1983). However, this study left the question open whether FOXO acts strictly in a cell-autonomous manner during aging. Subsequent studies of Drosophila FOXO showed that activation of FOXO in the adult adipose tissue is sufficient to extend lifespan, via reducing brain-derived DILP2 (Drosophila Insulin-Like Peptide 2) levels and insulin signaling in peripheral tissues (Giannakou et al., 2004; Hwangbo et al., 2004). This effect is mediated by FOXO-dependent transcription of *dilp6* in fat cells, which represses DILP2 secretion by neurosecretory cells (Bai et al., 2012).

Reduced protein homeostasis, a characteristic feature of aging, is associated with cellular decay over time due to insufficient protein quality control by the ubiquitin-proteasome system (UPS), by the autophagy/lysosome pathway, and by chaperone-mediated protein refolding (Lavandero et al., 2013; López-Otín et al., 2013). Protein quality control defects contribute to age-related muscle weakness, and FOXO and its transcriptional target Thor/4E-BP were found to enhance autophagy to remove damaged proteins. Moreover, *foxo* mutants exhibited proteostasis defects in their muscles. As muscle-specific overexpression of FOXO or 4E-BP promotes longevity and delays the functional decline of muscles with age, as well as modulates organism-wide proteostasis by affecting feeding behavior, this signaling route plays a critical role in the determination of lifespan (Demontis and Perrimon, 2010).

### The Insulin-PI3K-Akt Pathway

Similar to the previously mentioned *daf-2/insulin receptor* mutant in worms, reduced activity of this pathway led to longevity in Drosophila. One particular heteroallelic combination of hypomorphic insulin receptor gene alleles resulted in a 85% extension of maximal lifespan (Tatar et al., 2001). Mutation of *chico*, the single fly homolog of insulin receptor substrate proteins, caused a 48% extension of median lifespan in homozygous and 36% extension in heterozygous form (Clancy et al., 2001). Importantly, only homozygous mutants had a small body size, indicating that the effect of this pathway on cell/organismal growth is separate from its role in mediating longevity.

Interestingly, PI3K/Akt signaling is not only modulated by insulin and insulin-like growth factors. A recent study showed that mechanical stress is also necessary for the response of fat cells to insulin, both *in vivo* and *ex vivo* (Kim et al., 2018). This finding may explain how sedentary lifestyle (that is, reduced mechanical stress to tissues) contributes to insulin insensitivity.

The Activin-like TGF-β superfamily ligand dawdle (*daw*) was found to be repressed by FOXO during reduced insulin/insulin-like growth factor-1 signaling (Bai et al., 2013). Loss of *daw* or its downstream transcription factor *Smox* extended lifespan by preserving muscle function and reducing poly-ubiqutinated protein accumulation. It may not be FOXO that directly regulates the transcription of Atg genes because Smox was capable of binding and transcriptionally repressing *Atg8a* in *chico* mutants, and overexpression of *Atg8a* in muscles promoted longevity (Bai et al., 2013), similar to its neuronal overexpression (Simonsen et al., 2008).

### AMP-Activated Kinase

AMP-activated kinase (AMPK) is activated by low energy status manifesting in low ATP and high AMP levels or by its upstream kinase LKB1, which is activated by genotoxic stress and reactive oxygen species. AMPK not only controls glucose and lipid metabolism but also autophagy through direct phosphorylation of ULK1 (one of the mammalian homologs of Atg1 kinase) (Egan et al., 2011). Importantly, upregulation of AMPK activity in the adult Drosophila brain increased autophagy both in the brain and intestinal epithelium, and slowed systemic aging (Ulgherait et al., 2014). The upregulation of Atg1 in the brain was necessary and also sufficient for these effects. Similarly, upregulation of AMPK in the adult intestine induced autophagy both in cell autonomous and non-cell-autonomous manners and increased longevity. The tissue-specific AMPK/Atg1 activation was again found to reduce brain-derived DILP2 levels and organism-wide elevation of 4E-BP expression, this way slowing aging non-cell-autonomously (Ulgherait et al., 2014).

## Multiple Longevity-Inducing Paradigms Act Through Autophagy

Modulation of the previously discussed signaling pathways may help living a longer and healthier life. In this chapter we will discuss well-established options to potentially achieve this.

### Dietary Restriction

Dietary restriction (DR) extends the lifespan of multiple organisms including yeast, worms and flies, and it can slow down the development of many age-related diseases as well. DR is thought to induce a shift from growth and reproduction to somatic maintenance (Mair and Dillin, 2008). Importantly, food/calory intake must be reduced without causing malnutrition, which is often achieved by supplementation of the diet with essential nutrients. The main pathways that contribute to nutrient sensing and mediating longevity are TOR kinase and insulin/PI3K/Akt signaling, as mentioned before. The effect of DR on *chico* null mutant versus control flies was assessed under different food concentrations. Both genotypes showed peak lifespan with similar maximum values (55 days), however, *chico* nulls reach this at 0.8x diluted food concentration, while control lifespan was maximal at 0.65x dilution. The authors concluded that *chico* mutation did not further increase the longevity achieved by DR so these manipulations work through overlapping mechanisms (Clancy et al., 2002), but others recommended further analyses (Tatar, 2007).

Tor signaling is activated by nutrients and growth factors, so DR lowers TOR kinase activity and thus enhances autophagy. TOR also controls ribosomal biogenesis, mitochondrial function, and metabolism and thus its inhibition prefers a basal, “maintenance state” instead of growth, as reviewed in detail elsewhere (Kapahi et al., 2010). Apart from DR, TOR kinase activity can also be modulated pharmacologically as discussed below.

It is important to emphasize that DR can also be mimicked by mutations affecting nutrient transporters on the surface of cells. A well-known example for that is Indy (I’m Not Dead Yet), the fly ortholog of the plasma membrane transporter SLC13A5 (Rogina et al., 2000). Indy is expressed in tissues with high metabolic activity, and imports citrate and other TCA cycle intermediates. Mutations affecting *Indy* or its worm or mouse homologs extend lifespan and bring about metabolic and physiological changes that are also seen during DR (Rogers and Rogina, 2015). Promising small molecule inhibitors of Indy that are being developed may turn out to be effective in the treatment of age-related diseases (Willmes et al., 2018).

### Rapamycin

Rapamycin is an antifungal macrolide metabolite, which was discovered in a soil sample collected from Rapa Nui (Easter Island). It is produced by *Streptomyces hygroscopicus* and it has an immunosuppressive and anti-proliferative effect on mammalian cells (Li et al., 2014). Rapamycin and its analogs (rapalogs) are specific TOR inhibitors by engaging FKBP12 to bind to the FKBP12-rapamycin-binding (FRB) domain of TOR. This reduces the phosphorylation of two TORC1 effectors, S6K and 4E-BP. Dephosphorylated 4E-BP then shuts down eIF-4E mediated cap-dependent translation, while Atg1-dependent autophagy and cap-independent translation are upregulated (Ravikumar et al., 2004). Genetic inhibition of the Tor pathway promotes longevity in yeast (Kaeberlein et al., 2007), worms (Vellai et al., 2003), flies (Kapahi et al., 2004) and mice (Selman et al., 2009). Bjedov et al. (2010) investigated the effect of rapamycin on lifespan, stress resistance, fecundity and autophagy induction in Drosophila. Different concentrations of rapamycin were administered to the flies to find the effective concentration (50 μM) that gives maximal lifespan extension, which was independent of the TORC2-specific phosphorylation of Akt kinase and AMP-activated protein kinase (AMPK). TORC1 effector pathways were altered upon rapamycin feeding as expected: global translation was inhibited, and the number of LysoTracker-positive (acidic) lysosomes/autolysosomes increased suggesting autophagy induction. Rapamycin acted through autophagy based on experiments with *Atg5* RNAi flies, since its lifespan-elongating effect was abolished in knockdown animals. Moreover, rapamycin treatment increased stress resistance as it also prolonged the survival of flies that were either starved or treated with paraquat (a commonly used oxidative stress inducer). Unfortunately, pharmacological inhibition of human TOR kinase leads to immunosuppression so it is mainly used in organ transplant patients (Lamming et al., 2012), and as a result the potential use of rapamycin and newer generation TOR kinase inhibitors for lifespan extension therapy is limited.

### Exercise

Physical exercise has been implicated in healthy aging, which is especially critical considering the mostly sedentary life and high-calorie diet of modern men (Fontana, 2009). Exercise induces autophagy in skeletal and heart muscle, which is required for the systemic beneficial effects of training in mice (He et al., 2012). In line with this, combined strength and endurance training of senior people improves both neuromuscular and cardiorespiratory functions (Cadore et al., 2014). The effect of endurance exercise was also investigated in flies, and it promoted central and peripheral adaptations: improved cardiovascular function and capacity of skeletal muscles to generate energy via oxidative metabolism (Sujkowski et al., 2015). Genes that are upregulated after training in a machine called Power Tower (Tinkerhess et al., 2012) (developed for daily exercise of flies utilizing their negative geotaxis reflex) were identified and compared against genes that are associated with increased lifespan in a fly line generated through selective breeding. Genes coding odorant or gustatory receptors, members of the G-protein coupled receptor encoding methuselah-like family genes, lipases, enzymes in the folate metabolism and stress resistance were found to overlap between trained and long-lived flies. While no autophagy genes were identified in this study, enhanced lysosomal activity was seen in the adipose tissue of both long-lived flies and exercised control flies. These data suggest that autophagy is transiently upregulated in the muscle after exercise in flies as well (similar to mice), leading to stable upregulation of this process in adipose tissue.

### Resveratrol

Resveratrol is one of several polyphenols found in different plants such as grape berry skins, peanuts, blueberries and cranberries. Resveratrol and related agents were suggested to act as a Sirtuin activating compound (STAC), which promotes the deacetylation of proteins by members of the Sir2-like protein family. As the role of sirtuins in lifespan modulation is controversial (Madeo et al., 2019b), the effect of resveratrol is not clear either. A systematic study tested the effect of different concentrations of resveratrol on the lifespan of male and female flies raised on diets with variable amounts of fat, protein and carbohydrate, and only detected longevity in a small subset of conditions, and only in female flies (Wang et al., 2013).

### Antioxidants

Oxidative stress is thought to be one of the major drivers of the aging process. Both exogenous sources and mitochondria can produce reactive oxygen species (ROS) that then damage organelle membranes, proteins and other molecules inside cells, this way leading to a general malfunctioning over time. Autophagy may potentially circumvent the detrimental effects of oxidative stress via the enhanced turnover of damaged structures (Kourtis and Tavernarakis, 2011). In line with this model, expression of Ndi1 (a mitochondrial yeast NADH dehydrogenase) in Drosophila, which can act in parallel to mitochondrial complex I to reduce mitochondrial ROS production, promotes longevity (Sanz et al., 2010b). However, the direct causal relationship between oxidative stress and lifespan is not clear, because overexpression of the gene encoding alternative oxidase (AOX) decreases mitochondrial ROS production but does not increase lifespan, and *dj1-beta* mutant flies generate more ROS without detrimental effects on longevity (Sanz et al., 2010a).

### Spermidine

The polyamine spermidine affects the epigenetic deacetylation of histone H3 through inhibition of histone acetyltransferases (HAT). The intracellular concentration of spermidine decreases during aging, and the lifespan of yeast, worms, flies and in human immune cells can all be extended with the administration of spermidine (Eisenberg et al., 2009). Spermidine was taken up and metabolized by flies when their food was supplemented with this polyamine, and it increased their mean lifespan by 30%. Spermidine upregulated the transcription of autophagic genes, and increased the level of lipidated, autophagosome-associated Atg8a in flies and its homolog LC3 in human cells as well as the number of LysoTracker Red-positive (auto)lysosomes (Eisenberg et al., 2009).

The age-dependent decline of olfactory memory of Drosophila serves as a model for age-induced memory impairment (AMI). Decreased polyamine level in the heads of aging Drosophila was found to impair the olfactory memory of flies, because normal AMI could be restored with the administration of spermidine (Gupta et al., 2013). Spermidine enhances autophagy based on elevated Atg8a and decreased p62 levels observed in the brains of old flies on spermidine treatment. Autophagy is essential for mediating the effect of spermidine, since polyamine administration could not restore AMI in *Atg7* or *Atg8a* mutant flies (Gupta et al., 2013). Importantly, polyamines are candidate substances for treating not only AMI: ongoing clinical trials investigate the effect of spermidine-rich plant extracts on longevity (Kiechl et al., 2018; Schwarz et al., 2018).

### Additional Autophagy Enhancers That Potentially Promote Longevity

Metformin, a commonly used drug to treat type 2 diabetes, is a potent activator of AMPK and also a potential candidate lifespan extending and autophagy-inducing agent (Madeo et al., 2019b). Metformin has beneficial effects on the lifespan of mice (Martin-Montalvo et al., 2013). However, metformin failed to induce longevity in flies even though the treatment potently activated AMPK (Slack et al., 2012), suggesting that its effect may not be universal.

Non-steroid anti-inflammatory drugs are widely used in the clinics, and one of the most prevalent agents is aspirin. In line with this, aspirin was found to also promote longevity and healthspan in flies (Danilov et al., 2015; Song et al., 2017). As a relatively minor side effect, increased risk of gastrointestinal bleeding was reported earlier (Madeo et al., 2019b). However, a very recent study concluded that the use of low-dose aspirin was associated with an increased risk of intracranial hemorrhage, a major bleeding in the brain associated with a high risk of mortality (Huang W.Y. et al., 2019). Thus, preventive low-dose aspirin treatment can no longer be considered as low-risk.

## Conclusion

**FIGURE 1:**
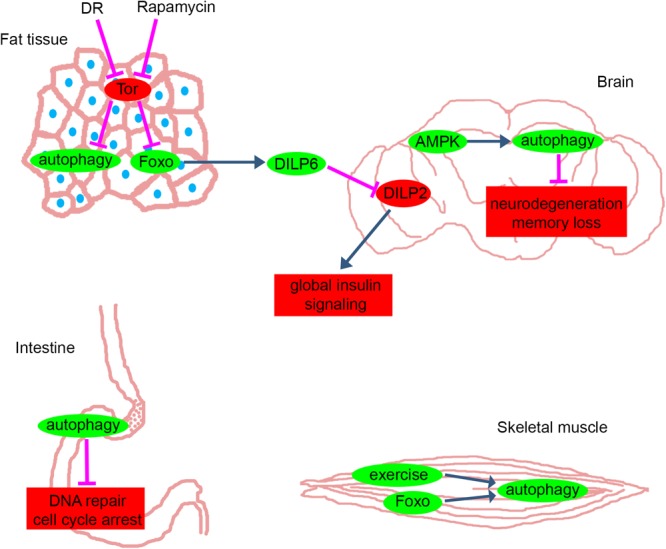
Anti-aging pathways in different tissues of Drosophila. The main pathways and proteins that counteract with aging in fat tissue, brain, intestine and skeletal muscle of Drosophila. Green: mechanisms and proteins that have anti-aging effects when they are activated or their levels are elevated. Red: mechanisms and proteins that have anti-aging effects when they are inhibited or their levels are decreased. Blue and magenta arrows represent activation and inhibition, respectively. DR, dietary restriction; Tor, Target of Rapamycin pathway; Foxo, forkhead box O transcription factor pathway; DILP, Drosophila Insulin-Like Peptide; AMPK, AMP-activated kinase. Please see text for further details.

Are we getting closer to finding the fountain of youth? While we understand several aspects of aging by now, we are far from having the whole picture. Various studies using Drosophila and other animal models show that several signaling pathways and interventions have well-established roles in the modulation of the aging process, as discussed in this review (summarized in Figure 1). Drosophila can be the first-line model to test hundreds of potential longevity-enhancers in a multicellular organism. Its advantage is clear as it suits well the investigation of cellular and systemic effects in a whole animal, and promising candidate treatments can be easily adapted to mammalian models. The conserved features of the main longevity pathways presume that the selected agents will have similar effects in mammals as well. New strategies and combinations of various agents that fine-tune autophagic activity can be selected based on Drosophila experiments. Increasing the specificity and efficacy of these autophagy-enhancers may also help controlling side effects.

One caveat of model animal studies is that human health and lifespan is strongly influenced by social and economic factors. Regardless of this major issue, we can certainly make general recommendations for a healthy lifestyle that includes regular exercise and permanent or intermittent caloric restriction (fasting), but these require serious efforts and engagement. It would be much easier to take some sort of food supplement, wouldn’t it? The potential use of caloric restriction mimetics discussed here and also in a recent review (Madeo et al., 2019b) may provide a solution. For example, it would be feasible to regularly eat spermidine-rich food, perhaps in combination with other agents that enhance autophagy and induce longevity, as their mechanisms of action are complex and only partially overlap (Madeo et al., 2019a).

## Author Contributions

TM, ZS-V, VK, TC, and GJ wrote the manuscript. TM, VK, and GJ prepared the figure.

## Conflict of Interest Statement

The authors declare that the research was conducted in the absence of any commercial or financial relationships that could be construed as a potential conflict of interest.
